# Occurrence and timing of complications following traumatic dental injuries: A retrospective study in a dental trauma department

**DOI:** 10.4317/jced.53022

**Published:** 2016-10-01

**Authors:** Shaul Lin, Nir Pilosof, Munir Karawani, Ronald Wigler, Arieh Y. Kaufman, Sorin T. Teich

**Affiliations:** 1 DMD, Endodontics and Dental Trauma Department, School of Graduate Dentistry, Rambam Medical Center, Haifa, Israel; 2 DMD, Faculty of Medicine at the Technion Institute of Technology, Haifa, Israel; 3 DMD, MBA, Case Western Reserve University School of Dental Medicine, Cleveland, OH, USA

## Abstract

**Background:**

This study explores the pattern of complications occurrence resulting from traumatic dental injuries, the relation of this pattern to the number of years from the time of the injury to its first diagnosis, and other contributing characteristics such as root development and trauma characteristic.

**Material and Methods:**

Patients’ data treated following dental trauma from 2002 to 2014 were classified and grouped according to age, gender, tooth type, injury type, diagnosis and the time that elapsed between the traumatic event and the diagnosis of complications (TIC). The distribution function of the quantitative parameters was determined with the Kolmogorov-Smirnov test. Fisher exact test was used to test differences between categorical parameters.

**Results:**

The review identified 166 patients (114 male and 52 female), with a total of 287 traumatized teeth, and a mean of 1.8 injured teeth per incident. Maxillary teeth were involved significantly more often in traumatic dental injuries. The follow-up period range (TIC) had a mean of 2.99 years. The most frequent complication was pulp necrosis (34.2%). The most frequent complication related to avulsion was ankylotic root resorption (50%) diagnosed after a median TIC of 1.18 years. Open apices at the occurrence of trauma were observed in 52 teeth. Of these, 54.9% experienced pulp necrosis and 9.8% inflammatory root resorption with a median TIC of 1.63 years. Teeth that experienced multiple traumatic events showed significantly more late pulp necrosis compared to teeth that experienced a single traumatic injury (61.9% vs. 25.3%, respectively, *p*<0.0001).

**Conclusions:**

Follow-up periods should be based on the type of traumatic dental injury and the severity of the potential complications for the tooth. Current recommendations for follow-up after traumatic dental injury should be revised to reflect the need for more frequent and overall prolonged follow-up.

** Key words:**Dental trauma, avulsion, open apex, pulp necrosis, root resorption, follow-up, complications.

## Introduction

Complications of injuries involving teeth and their supporting structures include pulp necrosis, ankylotic root resorption, inflammatory root resorption, and pulp canal obliteration (1-3). These may appear shortly after the trauma occurs or after a few years (4). The International Association of Dental Traumatology (IADT) and the American Association of Endodontics (AAE) guidelines (5-7) state that proper diagnosis, treatment planning, and follow-up care are critical to ensure a favorable outcome and recommend follow-up at 6-8 weeks and 1 year for events such as concussion and complicated crown fracture. For severe trauma, i.e., alveolar fracture and luxation injuries, more frequent follow-ups over a longer period are recommended at 4 weeks, 6-8 weeks, 4 months, 6 months, and once a year for up to 5 years (5-7). Most dental trauma guidelines, including these follow-up periods, are based on the literature, expert professional judgment and the consensus opinions (5-8).

Clinical studies describing the main complications that may affect traumatized teeth point to the importance of immediate treatment and seeking professional follow-up (9). Early diagnosis and treatment of traumatic injuries lead to better control of post-traumatic complications and to increased chances for conservation of the tooth and its surrounding structures (9,10). Long-term follow-up and monitoring are essential, especially in patients with a developing dentition, to avoid psychological and social impacts following dental trauma (11).

Although previous studies have described the assessment of pulp prognosis following tooth trauma (12-14), only a few have reported the relationship between complications and follow-up frequency (4,15,16). The status of apex development is another factor that seems to affect outcomes following dental trauma. The prevalence of pulp necrosis in traumatized teeth with complete root development is higher than those in which this process is incomplete (15,17).

This study explores the pattern of complications occurrence resulting from traumatic dental injuries (TDI), the relation of this pattern to the number of years from the time of the injury to its first diagnosis, and other contributing characteristics such as root development (apex status) and trauma characteristic (avulsed teeth).

## Material and Methods

This retrospective study was approved by the Institutional Review Board of the Rambam Medical Campus (#0051-13) and was conducted according to Harmonized Tripartite Guideline for Good Clinical Practice (ICH-GCP).

Medical records of patients who were treated at the Endodontics and Dental Trauma Department at the Rambam Medical Campus, Haifa, Israel from 2002 to 2014, were retrieved from the patient registry. All healthy patients treated at the department between the relevant years with at least 1-year of recorded follow-up were included. Exclusion was based on insufficient follow-up time, injury to deciduous teeth, or deficient records. The traumatic dental injury data were carefully screened, classified, and grouped according to age, gender, tooth type, injury type, diagnosis, and elapsed time between the traumatic event and the first diagnosis of complications. Patients were treated and followed according to the IADT recommendations time recall, (5,6) for their specific injury, depending on their compliance (clinical and radiographic examination included routine cold and electric pulp tester sensibility tests, percussion test, and mobility test), except for avulsion and intrusion cases, which were followed more frequently than recommended during the second year, i.e., every 6 months. Pulp necrosis was diagnosed by cold and electric pulp tests.

Statistical analysis was performed with SPSS version 21 (IBM Corporation, Armonk, NY, United States). The distribution function of the quantitative parameters (number of years from time of injury to first diagnosis of complications and age at the time of injury) was determined with the Kolmogorov-Smirnov test to determine the need to use non-parametric test. Fisher exact test was used to test differences between categorical parameters. A *p*-value of less than 0.05 was considered statistically significant.

## Results

**Figure 1:**
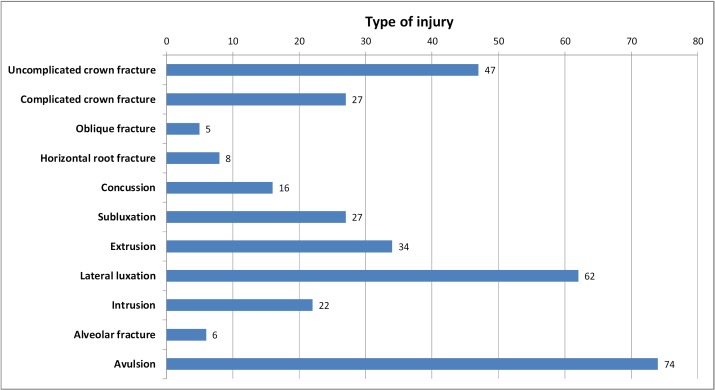
Number of teeth by injury type. Note- 254 teeth sustained one injury, 42 teeth sustained more than one injury type.

The review identified 166 patients (114 male and 52 female), with a male-to-female ratio of 2.2:1, having a total of 287 traumatized teeth with a mean of 1.8 injured teeth per incident. The average patient age was 14.34±10.01 years and ranged from 6-69 years. Maxillary teeth were involved significantly more often in traumatic dental injuries, the most involved teeth being the maxillary central incisors (34.2% of all injuries were related to tooth 11 and 36.9% to tooth 21), followed by the maxillary lateral incisors (8.1% related to tooth 12 and 9.5% to tooth 22 – teeth numbering according with the FDI numbering system). Figure 1 summarizes the types of injuries recorded for this report. Uncomplicated crown fracture (N=47 teeth), lateral luxation (N=62 teeth), and avulsion (N=74 teeth) were the most common injuries.

Distribution analysis of the period from injury to first diagnosis of complications and patient’s age at the time of injury was determined with the Kolmogorov-Smirnov test (*p*<0.001 for both parameters); based on these results, non-parametric tests were used for statistical analysis.

The follow-up period range from the time of injury to the first diagnosis of complications (TIC), was 1-12 years, with a mean of 2.99 years. The frequency of occurrence of the different complications is shown in  Table 1. The most frequent complication was pulp necrosis (34.2%), which was categorized as either “early pulp necrosis” (3.5%) (occurring within the first 3-month follow-up period), or “late pulp necrosis” (30.7%). For each type of complication, the TIC was compared to the TIC of all other traumatized teeth that did not experience a similar complication. For example, the median TIC for early pulp necrosis was 0.09 years and the TIC for all other teeth was 1.30 years. Mann Whitney U test revealed that this difference was significant (*p*<0.001). Significant differences also were observed in regard to root canal obliteration and cervical resorption (*p*<0.001 and *p*=0.017, respectively).

**Table 1:**
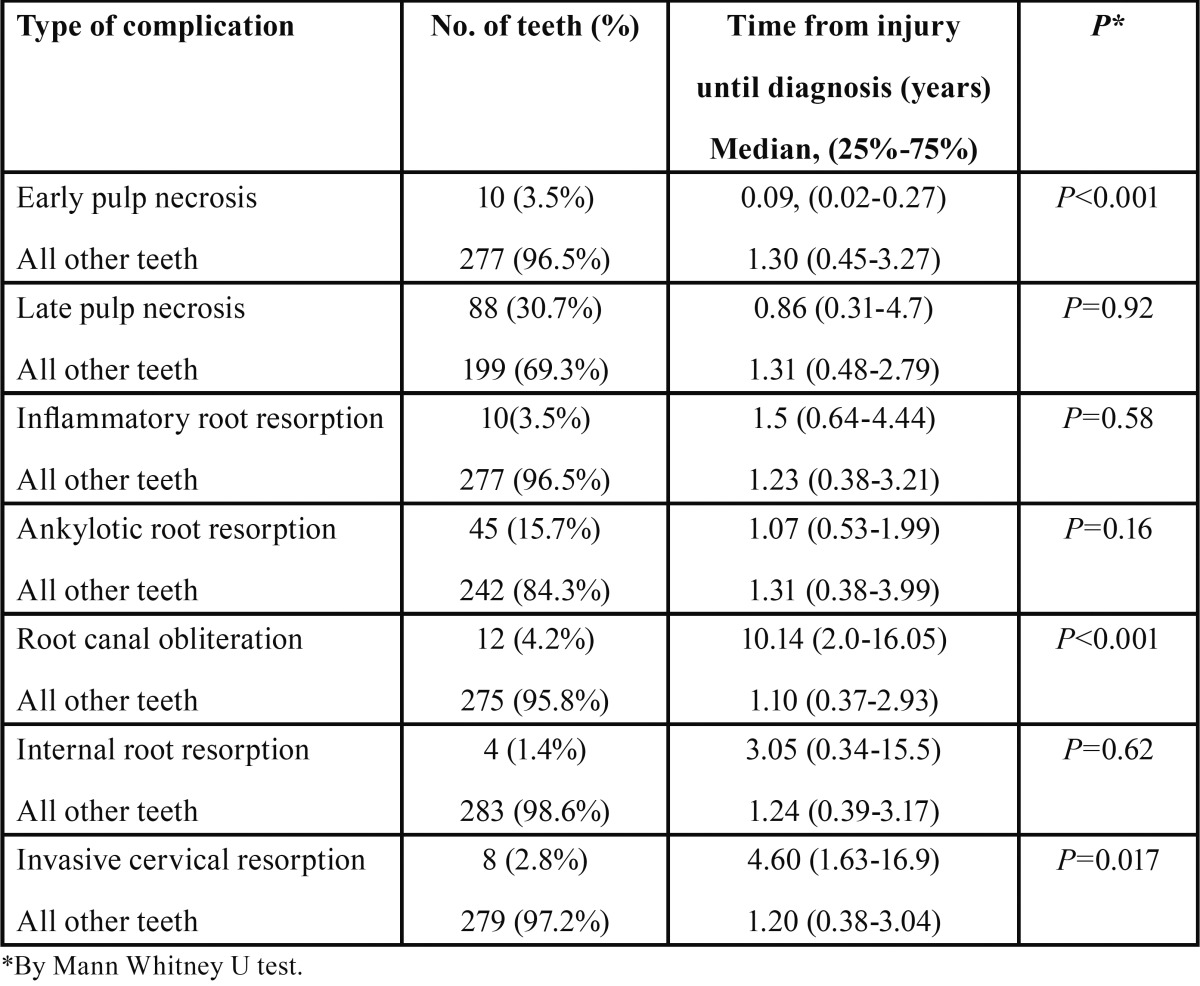
Complications following Traumatic Dental Injuries (TDI) – Time from injury until diagnosis (TIC).

The same type of comparison was done in regard to the age at the time of injury ( Table 2). Patients who experienced cervical resorption were older than those who had injuries but did not display this complication (*p*=0.020).

**Table 2:**
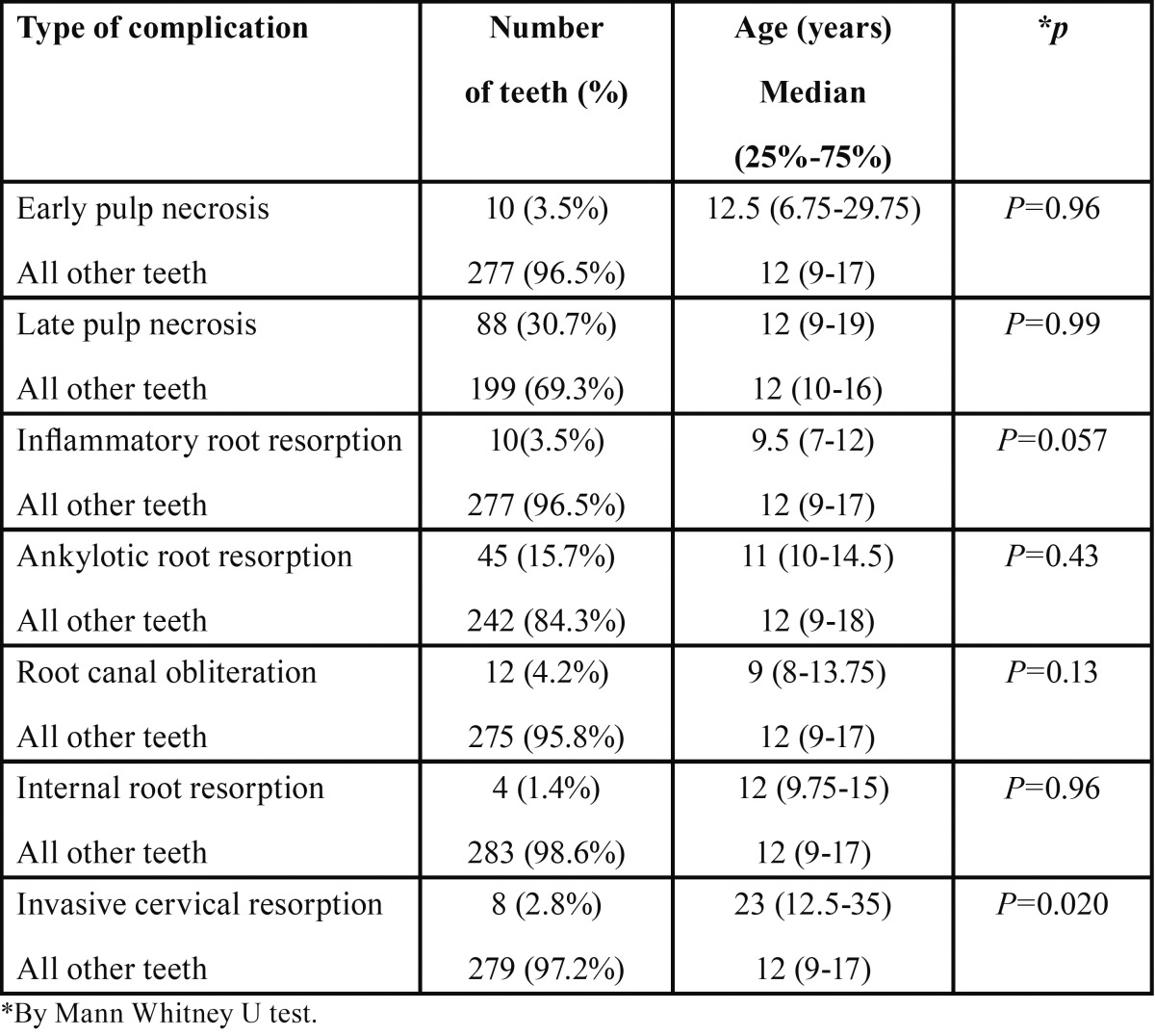
Complications following Traumatic Dental Injuries (TDI) – Patient’s age at the time of injury.

Similarly, 74 avulsion cases were analyzed separately.  Table 3 summarizes the frequency of complications following an avulsion injury, the TIC, and the age at injury. The most frequent complication related to avulsion was ankylotic root resorption (50%) that was diagnosed by X-ray. Ankylotic root resorption was diagnosed after a median TIC of 1.18 years that was a significantly shorter follow-up period compared to avulsed teeth that did not manifest this complication (*p*=0.039).

**Table 3:**
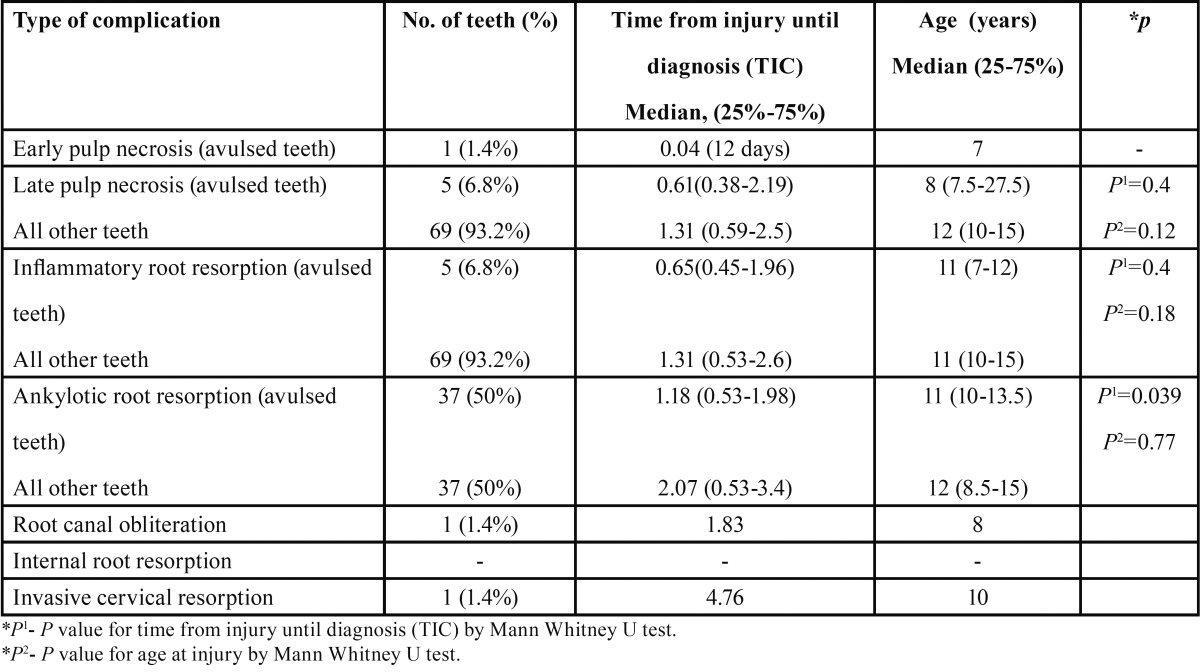
Complications following avulsion (N=74 teeth-- 25.8% from overall trauma cases).

According to IADT recommendations, proper storage for teeth for more than 60 minutes includes a physiologically or osmotically balanced medium. Complications following avulsion cases were further analyzed with regard to the storage medium and the time the tooth was kept extra-orally ( Table 4). Ankylotic root resorption was the most common complication for both properly stored teeth with less than 60 minutes dry time before replantation (37.8%) and improperly stored teeth or teeth kept dry longer than 60 minutes (69%) (*p*=0.017), with the median period from injury to first diagnosis being 1.24 years and 0.97 years, respectively (*p*=0.15).

**Table 4:**
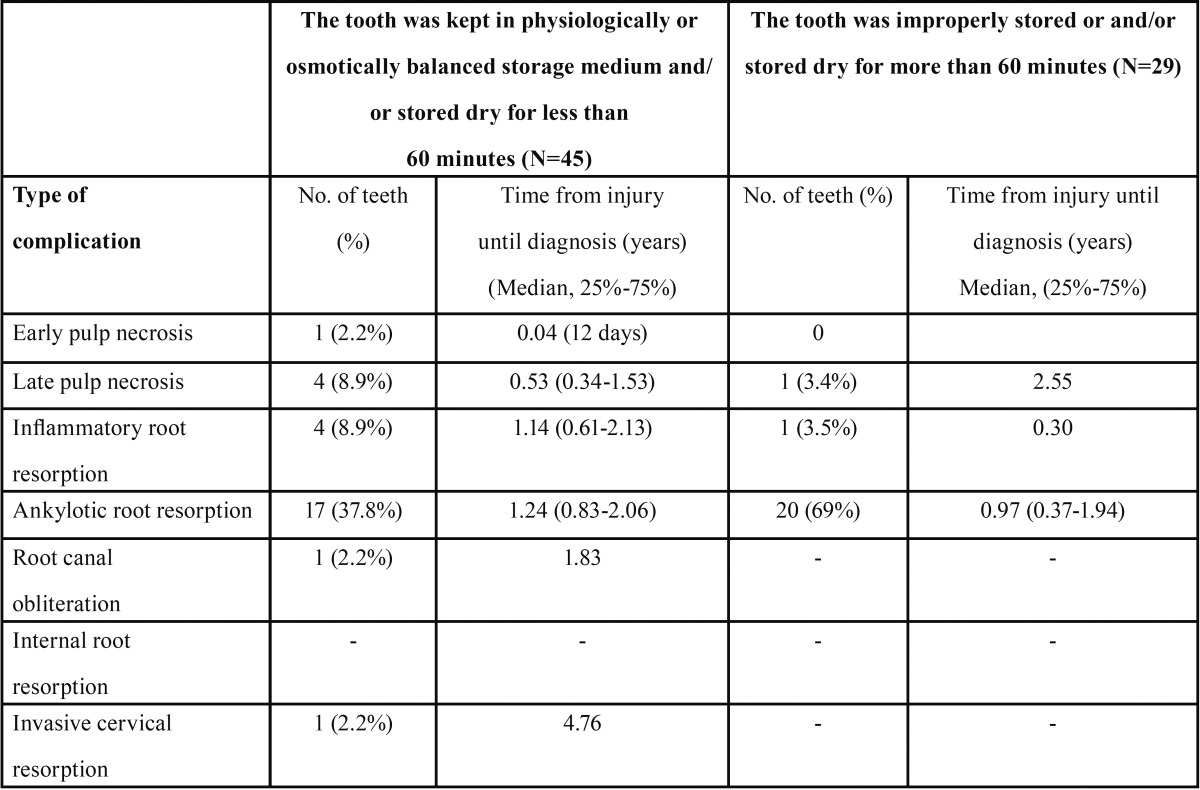
Complications following avulsion - according to the way teeth were stored after injury.

Of the 287 teeth examined, 52 (18.1%) had open apices at the time of occurrence of the trauma ( Table 5). Only 7.8% of teeth with an open apex experienced early pulp necrosis, whereas 47.1% manifested late pulp necrosis with a median for first diagnosis of 1.00 year after the trauma. Radiographic signs of inflammatory root resorption occurred in 9.8% of teeth with an open apex, and had a median first diagnosis time of 1.63 years post-trauma. Fisher Exact Test revealed that late pulp necrosis and inflammatory pulp resorption were significantly more prevalent (*p*=0.007 and *p*=0.018, respectively) in teeth that experienced trauma while having an open apex, compared to teeth that had a closed apex at the time of the injury ( Table 6).

**Table 5:**
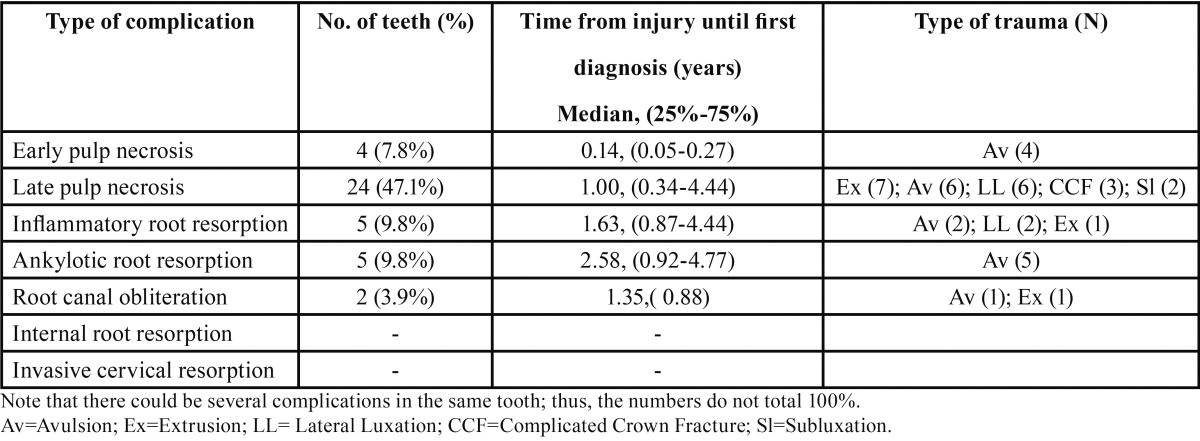
Complications in teeth with open apices (N=51) and the time elapsed until they were first diagnosed.

**Table 6:**
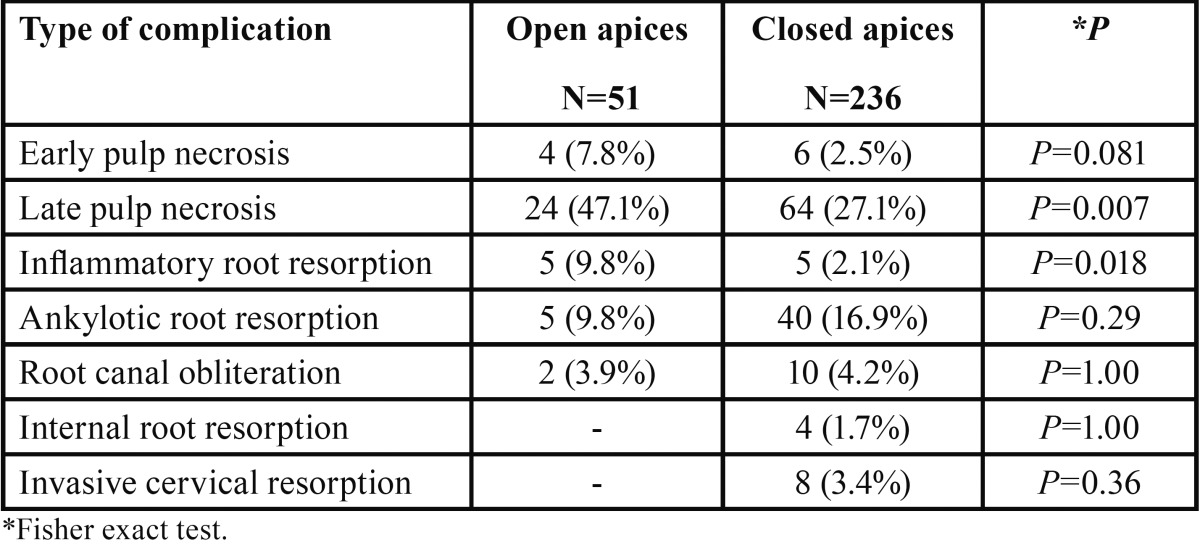
Complications in teeth that experienced trauma while having an open or close apex.

Teeth that experienced multiple traumatic events experienced significantly more late pulp necrosis compared to teeth that experienced a single traumatic injury (61.9% vs. 25.3%, respectively, *p*<0.0001) ( Table 7). Ankylotic root resorption was observed in 17.6% of teeth with single trauma and in 4.8% of teeth with multiple traumas (*p*=0.038). Out of a total of 45 teeth that displayed ankylotic root resorption, 37 teeth experienced this complication as the result of avulsion ( Table 3).

**Table 7:**
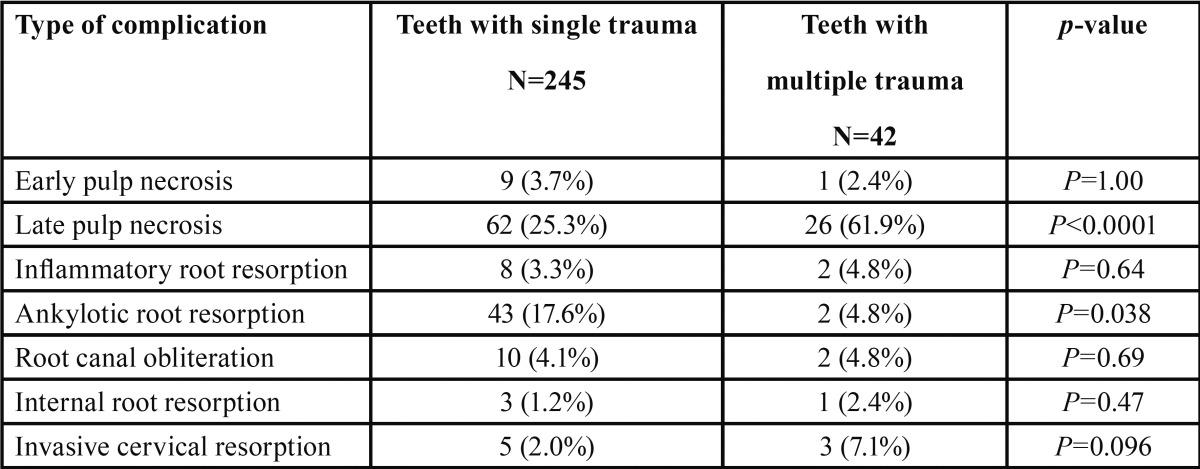
Complication rates in teeth with single and multiple traumatic events.

## Discussion

Traumatized teeth show a spectrum of responses, which range from no lasting effects to teeth that ultimately are not restorable. In the present study the follow-up period of traumatized teeth ranged from 1-12 years, with a mean of 2.99 years. Pulp necrosis was a frequent complication occurring in 34.2% of the cases, with most cases categorized as late necrosis that manifested years after the injury occurred ( Table 1). The prevalence of pulp necrosis in our sample is similar to what previously reported for traumatized teeth (18). Andreasen & Pedersen (19) studied the development of pulp necrosis following dental trauma and showed that pulp necrosis in mild injuries such as concussion could appear within 3 months, while after severe trauma such as lateral luxation and intrusion it will typically be manifested after nearly 2 years. Furthermore, the risk of pulp necrosis increased with the extent of the injury. For example, concussion and subluxation generated the least risk (3% and 6%, respectively), while lateral luxation and intrusion have the greatest risk of pulp necrosis (19). These results and other reports (18,20) are aligned with our findings that show most pulp necrosis to occur within the first year after trauma. The high number of cases of pulp necrosis suggest the importance of sealing exposed dentinal tubules after the trauma. The quality of the bacteria-tight seal provided by enamel-bonded resin restorations is an important factor in the success of pulp survival (21).

Root canal obliteration was reported to occur in 35% of traumatized teeth and to be related to the degree of extrusion (22) and the degree of root development (23). Other authors, however, despite finding similar rates of obliteration did not find the same relation (20). In our sample, root canal obliteration was diagnosed after 10 years (median value) of follow-up and had a relative low prevalence of 4.2% compared to a previous study that reported this complication occuring in 9% of the traumatized teeth ( Table 1) (18). Teeth with invasive cervical resorptions were diagnosed after more than 4.5 years following the injury. This type of root resorption was significantly more prevalent in patients who were older (*p*=0.020) at the time of the traumatic injury ( Table 2). As stated by Heithersay (24), trauma is one of the major predisposing factors for invasive cervical resorption and, because the prognosis is dependent on the stage of the resorption, early detection is crucial. This illustrates the importance of long-term follow up for traumatized teeth to catch these late-developing complications.

Andreasen *et al.* (4) found that complications such as inflammatory root resorption and ankylotic root resorption usually were radiographically diagnosed within the first 2-3 years after avulsion. In our study, inflammatory root resorption occurred only in 6.8% of the avulsed teeth and the median diagnosis time was one year post-injury ( Table 3). This result is lower than a previously published study that reported inflammatory root resorption occurring in 26.5% of replanted teeth (18). This may be related to the relatively large number of teeth that were kept in adequate conditions between the traumatic event and replantating ( Table 4). Ankylotic root resorption was the most prevalent complication of teeth re-implanted after avulsion (50% of affected teeth), a percentage consistent with a previous report (18). This complication occurred significantly earlier compared to non-avulsed teeth that exhibited this type of resorption (1.18 years vs. 2.07 years, *p*=0.039). As expected (18), out of a total of 45 teeth that displayed ankylotic root resorption, 37 teeth experienced this complication as the result of avulsion. These results suggest that follow-ups once a year, from the second year and for up to 5 years, are insufficient, and more frequent follow-ups should be performed following teeth avulsion. The first radiographic signs of ankylotic root resorption appeared 1.24 years after TDI in teeth that were kept in physiologically or osmotically balanced storage medium for less than 60 minutes compared to 0.97 years for teeth that were not kept in ideal conditions ( Table 4), this time difference being not statistically significant. However, if the tooth was kept in an osmotically balanced medium or stored dry for less than 60 minutes, this type of resorption occurred in only 37.8% of teeth, whereas in instances in which the teeth were improperly stored this complication was observed in 69% of the cases. Despite the poor prognosis for replantation of immature and mature teeth that had not been kept in proper storage medium or stored dry for less than 60 minutes, replantation is recommended for aesthetic, functional, and physiological reasons, including the maintenance of the alveolar bone contour (25-27). Ankylotic root resorption, especially in young, growing children, has significant clinical implications, such as functional and aesthetic changes, that require treatment by a multidisciplinary team of specialists (25,28,29). This complication is frequently associated with infra-position, before or during growth spurts in children and adolescents, and decoronation may be necessary when an infra-position of more than 1 mm is detected (28-30).

Of the 287 teeth examined, 51 (17.8%) had open apices ( Table 5). The most common complication in teeth with an open apex was late pulp necrosis (47.1%), and the median time from injury to first diagnosis was one year. Early pulp necrosis occurred in additional 7.8% of injured teeth. It is interesting to note that in our cohort, teeth with open apices developed significantly more late pulp necrosis and subsequent inflammatory root resorption (*p*=0.007 and *p*=0.018, respectively,  Table 6). Previous studies determined that teeth with completed root formation showed a greater risk of pulp necrosis than teeth with incomplete root formation (17-19), but were consistent with our results in regard to the relation between increased occurrence of inflammatory root resorption in teeth with open apices (18).

Studies on combination injuries and the risk of pulp necrosis in permanent teeth show that a concomitant crown fracture, without pulp exposure, significantly increases the risk of pulp necrosis in teeth with concussion, subluxation and lateral luxation (1-3). These findings agree with our results, which show that pulp necrosis occurring in 61.9% of teeth that were subjected to multiple trauma events. This rate is significantly higher (*p*<0.0001) than the occurrence of pulp necrosis in teeth that experienced only one traumatic injury (25.3%) ( Table 7). Only two instances of ankylotic root resorption were found in our sample of teeth that had sustained more than one traumatic injury (N=42 teeth), a significantly lower rate than observed in teeth with only one TDI (*p*=0.038). This result may be attributed to the fact that most ankylotic root resorptions (N=37) were associated with avulsion cases that were not associated with secondary trauma events.

Follow-up periods should be based on the type of traumatic dental injury and the severity of the potential complications for the tooth. An in-depth analysis of the long-term complications and the time of their occurrence will help improve patient care and optimize the protocols for the follow-up period. Current recommendations for follow-up after traumatic dental injury that range from 4 weeks to 5 years, depending on the trauma characteristics (5-7) should be revised to reflect the need for more frequent and overall prolonged follow-up.
